# Ophthalmology

**Published:** 1896-03

**Authors:** F. R. Cross


### OPHTHALMOLOGY.

At the recent Congress of Oculists at Heidelberg, Dr. Hori, of Japan, described[5] a case of **hepatic amblyopia.** A man, aged 40, suffering from jaundice and diarrhœa, due to a neoplasm in the bile-ducts, developed colour-blindness for red and green, with general amblyopia. No perceptible ophthalmoscopic changes in the fundus could be detected. Septic keratitis, and dryness with atrophy of the conjunctiva supervened. After death an œdema of the retina was discovered, which accounted for the amblyopia. Prof. Leber stated that he had met with several cases of hemeralopia—defective vision by dull light—in jaundice, which he had attributed to retinal œdema, with impaired secretion or increased destruction of the retinal purple. Dr. Meyer mentioned cases of malarial fever with hepatic affections associated with hemeralopia, particularly among colonists; he had found pigmentation and other changes in the walls of the retinal arteries. It would be well to examine the liver in cases of hemeralopia, even where no jaundice is present, as the eye symptoms may be the earliest indications of hepatic defect.

[1] *Ann. Surg.*, 1894, xix. 47. [2] *Ibid.*
[3] *Am. J. M. Sc.*, 1896, cxi. 67.
[4] *Brit. M. J.*, 1892, i. 262. [5] *Med. Week*, 1895, iii. 398.

**Detachment of the retina** is undoubtedly a very rare **sequel of albuminuric retinitis**, but at one of the meetings of the Clinical Society of London[1] Dr. Samuel West mentioned two cases. (1) A woman, aged 22, presented the usual symptoms of granular kidney with retinitis. The nephritis had commenced seven years previously, in an acute attack. Shortly after her admission to the hospital a general purpura appeared, but lasted only a few days. After it both retinæ became detached, apparently not as a consequence of hemorrhage, though a large and very painful post-ocular hemorrhage occurred about the same time. (2) A young man, with granular kidney, who had been apparently in good health until two months previously. His sight had been complained of for a week only, but he was admitted to hospital practically blind ; the eyes showed a marked degree of albuminuric retinitis. Seventeen days later detachment of the left retina was discovered, for which no special cause seemed apparent. A few days later he died of diarrhœa and asthenia.

The opinion appears to be universally held that the appearance of retinal complications in albuminuria makes it probable that the disease is in an advanced stage and will be rapidly fatal. Where the retinitis is complicated by detachment of the retina, or by hemorrhages of a purpuric nature, the prognosis would probably be increasingly grave.

I have seen, in two cases, a very large **vitreous hemorrhage** complicate retinitis **with albuminuria.** One of these was a woman of 50, who showed well-marked retinitis with hemorrhage and œdema apparently of albuminuric type, but no albumen could be discovered in the urine, which was of low specific gravity, until several weeks after she first came under observation. Before albumen was detected by chemical examination, the fundus reflex of one eye became completely hidden by a vitreous hemorrhage, which appeared to occupy the whole chamber. Meanwhile albumen appeared in the urine, and the patient's condition became precarious from dropsical effusions. Improvement gradually occurred, the albumen disappeared, the synchysis of the vitreous cleared so that the eye fundus could be seen in some detail, while the retinitis of the other eye almost entirely cleared up, very few small hemorrhages alone remaining. This case shows that the appearances of retinal complications in albuminuria may again disappear, and that such retinal complication does not always mean that the case will be rapidly fatal. Dr. Hale White, at the meeting of the Clinical Society already alluded to, related a case in which, despite the continuance of Bright's disease, well-marked retinitis had become completely resolved—he is of opinion that interstitial nephritis is rapidly fatal under thirty years of age.

The association of retinitis with albuminuria in pregnant women demands early recognition and immediate delivery of the

[1] *Tr. Clin. Soc. Lond.*, 1895, xxviii. 100.

child, and while delay of interference may result in blindness from a ruined retina, if not in death from puerperal eclampsia, a timely delivery is generally followed by complete recovery both of the kidneys and of the eyes.

The serious prognosis of albuminuric retinitis is largely due to the tendency of an analogous vascular breakdown in the circulation of the brain, and death from cerebral hemorrhage is the not infrequent sequel of cases where the symptoms of the disease produce no very great discomfort, either in the sight or in the general health.

**Disorganisation of the vitreous with synchysis** appears but seldom materially to improve. Abadie made an interesting communication to the Paris Academy of Medicine[1] of the good result of electrolysis in such cases. A patient shown to the members had, eighteen months before, during an uncontrollable fit of anger, begun to lose his sight, and in a space of two or three hours was practically blind. His eyes were not examined at this time, but eight months afterwards the vitreous body of each was found to be disorganised, probably from intraocular hemorrhage. Some months later Abadie punctured the left eye by a fine iridised platinum needle 8 mm. long. This needle was connected with the positive pole of a continuous current battery, the negative pole of which was applied to the arm. A current of three or four milliampères was passed through the circuit for five minutes. The following day the patient commenced to count fingers, and the fundus of the eye, till then quite obscure, could be illuminated by the ophthalmoscope. Gradual improvement continued. At the end of a month the patient could go about alone, could read the names of streets and the number of the houses. The right eye, which had not been treated, remained in a sightless condition with dark and opaque vitreous. Abadie states that he has since similarly treated an analogous case, in which the vitreous was disorganised by successive hemorrhages. This also was cured.

At the Medical Society of Buda-Pesth[2] Dr. Grosz reported a case of **amaurosis produced by male fern.** A man, aged 29, had been given by a pharmacist capsules containing ethereal extract of male fern and extract of pomegranate. He took about eight grammes of the combined medication, after previously taking castor oil. Hitherto vision had been perfect in the right eye, somewhat weak in the left. In the evening he had syncope, the next day severe diarrhœa, and two days later he was blind in both eyes. On examination there was complete mydriasis, no pupil reaction to light. At first the fundus of each eye was normal, but little by little both lost their colour, and became

[1] *Ann. d'Ocul.*, 1895, cxiv. 134. [2] *Ibid.*, 207.

atrophic. The noxious influence was attributed to the extract of male fern, which is toxic in doses of four to five grammes.

Prof. Masius reported to the Academy of Medicine of Belgium [1] the results of some experiments on dogs, made to ascertain whether the amaurosis, which he had also noticed in two cases to follow the administration of oleoresin of male fern for ankylostomata, was due to the anæmia caused by the worms, or to the remedy used to destroy them. Of four dogs two rapidly became blind after taking large doses of the male fern. In a later communication Masius [2] gave the result of researches he had made with Dr. Mahaim on the anatomical lesions of the optic nerve produced in poisoning by the oleoresin of male fern. The primary lesion is vascular, and consists in proliferation of capillaries with cell infiltration of the perivascular space. This determines at a very early date strangulation of the optic nerve in the foramen opticum; later on nothing is found at this spot but a homogeneous mass constituting a thick wall for the capillaries. Further advance shows disappearance of the nerve fibres, the last ones to persist being those situated near the retina.

Poulson of Strasburg has lately shown that among the extractives from male fern, anhydrous crystalline filicic acid is neither a poison nor a vermifuge, while amorphous filicic acid possesses both these properties in a high degree. Van Aubel of Liége [3] attributes the amaurosis to filicic acid, which stimulates the central nervous system, especially the spinal cord, and may, by extending to the sympathetic system, cause dilatation of the pupils, stimulation of the vasomotor nerves, and contraction of the central arteries of the retina; on this hypothesis strychnine and nitrite of amyl might be useful remedies.

Dr. Hilbert,[4] a German physician, has had under observation a middle-aged man, who to cure headache took a gramme and half an hour later two grammes of **acetanilide** (antifebrin). The headache soon ceased, but the patient was affected with vertigo, noises in the ears, and defective vision, so that he could barely see fingers at a foot. The skin and mucous membranes were pale. There was no reaction of the pupils, the optic discs were bloodless, the retinal vessels were contracted: all these phenomena, including amblyopia, yielded promptly to inhalations of amyl nitrite.

For purposes of accurate ophthalmoscopic examination a **mydriatic** is often necessary: and patients are caused considerable discomfort by the somewhat prolonged effect of nearly all drugs that produce sufficient dilatation of the pupil. The effect of homatropine lasts for, at any rate, a day, while atropine causes inconvenience for upwards of a week. Cocaine is scarcely

[1] *Med. Week*, 1895, iii. 321.

[2] *Ibid.*, 585. [3] *Ibid.*, 479. [4] *Ibid.*, 430.

active enough. Dr. Groenouw has recently suggested[1] the following solution:

| | |
|---|---|
| Hydrochlorate of Ephedrin ... | 1 part. |
| Hydrochlorate of Homatropine | $\frac{1}{100}$ ,, |
| Distilled Water ... ... ... ... ... | 10 parts. |

This is said to produce a maximum pupil-dilation in half-an-hour, and to leave no effects after one hour. The transient mydriasis is not associated with any change of the ciliary muscle.

F. R. Cross.

